# The broader economic impact of vaccination: reviewing and appraising the strength of evidence

**DOI:** 10.1186/s12916-015-0446-9

**Published:** 2015-09-03

**Authors:** Mark Jit, Raymond Hutubessy, May Ee Png, Neisha Sundaram, Jananie Audimulam, Safiyah Salim, Joanne Yoong

**Affiliations:** Department of Infectious Disease Epidemiology, London School of Hygiene and Tropical Medicine, Keppel Street, London, WC1E 7HT UK; Modelling and Economics Unit, Public Health England, 61 Colindale Avenue, London, NW9 5EQ UK; Initiative for Vaccine Research, World Health Organization, 20 Avenue Appia, 1211, Geneva, 27 Switzerland; Saw Swee Hock School of Public Health, Tahir Foundation Building, National University of Singapore, 12 Science Drive 2, Singapore, 117549 Singapore

**Keywords:** Cost-effectiveness, Health economics, Immunisation, Systematic review, Vaccines

## Abstract

**Background:**

Microeconomic evaluations of public health programmes such as immunisation typically only consider direct health benefits and medical cost savings. Broader economic benefits around childhood development, household behaviour, and macro-economic indicators are increasingly important, but the evidence linking immunization to such benefits is unclear.

**Methods:**

A conceptual framework of pathways between immunisation and its proposed broader economic benefits was developed through expert consultation. Relevant articles were obtained from previous reviews, snowballing, and expert consultation. Articles were associated with one of the pathways and quality assessed using modified Grading of Recommendations Assessment, Development, and Evaluation (GRADE) criteria.

**Results:**

We found 20 studies directly relevant to one or more pathways. Evidence of moderate quality from experimental and observational studies was found for benefits due to immunisation in improved childhood physical development, educational outcomes, and equity in distribution of health gains. Only modelling evidence or evidence outside the immunization field supports extrapolating these benefits to household economic behaviour and macro-economic indicators.

**Conclusion:**

Innovative use of experimental and observational study designs is needed to fill evidence gaps around key pathways between immunisation and many of its proposed economic benefits.

**Electronic supplementary material:**

The online version of this article (doi:10.1186/s12916-015-0446-9) contains supplementary material, which is available to authorized users.

## Background

Investment in immunisation programmes in both developed and developing countries has dramatically increased over the past two decades [1]. This is partly due to the development of new vaccines against major diseases [2], as well as the growth of new financing mechanisms through organisations such as Gavi, the Vaccine Alliance, and the Pan American Health Organization [3]. Spending growth has, in turn, heightened the importance of rigorous justification of the value of investing in immunisation [4]. Microeconomic evaluations are used to inform decision-making by national and multinational stakeholders, by comparing the economic cost of implementing vaccine program infrastructure, purchase, and delivery, against the health and economic benefits of vaccination.

However, many cost-effectiveness studies only consider direct health benefits and medical cost savings, although some consider a few wider benefits such as indirect (herd) protection and care-related productivity gains. However, economists have argued that improvements in health lead to economic growth through longer-term mechanisms such as decreasing fertility, strengthening macroeconomic stability, and improving educational outcomes [5, 6]. More recently, this economic theory has been applied to investments in immunisation. Bärnighausen et al. [7, 8] suggested that the benefits of immunisation programmes could be divided into ‘narrow’ and ‘broad’ benefits. Gains in health, health care costs, and care-related productivity typically considered in microeconomic evaluations were categorised as ‘narrow’ benefits, while additional benefits not normally incorporated were categorised as ‘broad’ benefits. Other authors have also proposed pathways by which investment in improving child health can reap benefits not captured in many evaluations [5, 6]. Previous reviews have focused on enumerating and categorising the proposed benefits, rather than appraising the strength of evidence behind each of them. Several taxonomies describing these putative benefits have been proposed [9, 10], and the literature describing them has recently been reviewed [10, 11]. However, the degree to which the claims of broader benefits are evidence-based is not clear.

To address this gap, we constructed a conceptual framework that captures causal pathways linking vaccines to their proposed benefits and conducted a rapid review of the validity and strength of evidence behind each pathway. Our aim is to inform research into filling knowledge gaps in this area, as well as to strengthen evidence-based advocacy and decision making about vaccination.

## Methods

### Analytical framework

Two authors of this report (MJ and RH) facilitated expert consultations during vaccine meetings convened by the World Health Organization in Toronto (13–14 July 2011), Geneva (28–29 June 2012), Sydney (10–11 July 2013), and Bangkok (24–25 November 2014; see Additional file 1 for participants). Experts at these meetings were given recent commentaries on the broader economic impacts of vaccines and immunisation programmes, and asked to propose conceptual frameworks mapping the potential causal relationships between immunisation and these impacts. Formulating the framework took part in several stages (1) a wide ranging discussion that was synthesized in the form of an initial flow diagram that participants agreed on (Toronto), (2) further expansion of the flow diagram in an open discussion, as well as formulating detailed descriptions of each benefit category (Geneva), (3) final refinement of the pathways (Sydney), and (4) discussion of preliminary results of the evidence review (Bangkok).

### Review

Articles describing evidence about the broader economic benefits of vaccination and immunisation programmes were reviewed. Because the scope of these benefits is poorly defined, a systematic review broad enough to encompass all potential benefits would need to cover almost the entirety of the vaccine literature. However, several more targeted reviews of such benefits have recently been conducted. Rather than to repeat the search process, we opted to identify relevant studies from four existing reviews [10–13], of which two were systematic [10, 11]. Further references were identified by the ‘snowball’ method (identifying relevant studies in the reference lists of articles already included), and from the expert consultations described previously.

We only reviewed articles for evidence about causal pathways to broader economic impacts (B3–D4 in Table 1). We define causality as meaning: (1) vaccination under a given set of conditions inevitably produces the relevant benefits, and (2) without vaccination the given set of conditions on its own does not inevitably produce the relevant benefits. We did not assess evidence supporting the association between vaccination and reduced morbidity/mortality in vaccine recipients, averted health care expenditure, or productivity losses directly experienced as a result of illness episodes. This is because there are numerous trials and trial-based economic evaluations in the epidemiological (as opposed to economic) literature that were beyond the scope of the reviews we used, but which give robust experimental evidence about reductions in morbidity, mortality, and direct and indirect healthcare costs as a result of vaccination (see for example [14, 15]). Similarly, we did not assess the quality of evidence behind herd immunity following vaccination, since this is again well-established from a multitude of cluster randomized, household randomized, observational, and post-licensure surveillance studies (see for example [16, 17]).

**Table 1:** Detailed descriptions of each proposed benefit of immunisation programmes

Category	Definition	Outcome measures
A. Health-related benefits to vaccinated individuals		
A1. Health gains	Reduction in morbidity and mortality	Cases averted
		Deaths averted
		QALYs/DALYs saved
A2. Health care cost savings	Reduction in direct cost of health care borne by the public sector or private individuals	Costs saved by health care provider
		Health care costs saved by individuals
B. Productivity-related benefits		
B1. Productivity gains related to care	Reduction in lost days of work due to caring for a sick patient	Value of productivity
B2. Productivity gains related to health effects	Reduction in lost days of work due to sickness or death of a sick patient	Friction costs
		Potential lifetime earnings
B3. Productivity gains related to non-utility capabilities^a^	Increased lifetime productivity because of enhanced capabilities (such as improved cognition and educational attainment) not easily measured using utility-based preference measures	Educational outcomes
		Cognitive outcomes
		Potential lifetime earnings
C. Community or health systems externalities		
C1. Ecological effects	Health improvements in unvaccinated community members as a result of ecological effects such as herd immunity, eradication, and reduced antibiotic usage	Indirect vaccine protection
		Prevalence of antibiotic resistance
		Future cost of disease control averted
C2. Equity	More equal distribution of health outcomes	Distribution of health outcomes
C3. Financial and programmatic synergies and sustainability	Improved financial sustainability as a result of effects such as synergies with other health care programmes (e.g. delivery platforms), stimulation of private demand, and mechanisms to enhance group purchasing power (e.g. PAHO revolving fund)	Financial benefits
		Private demand estimates
C4. Household security	Improved financial security of households as a result of reduced risk of catastrophic expenditure	Actuarial value of security
D. Broader economic indicators		
D1. Changes to household behaviour	Economic improvements due to changes in household choices such as fertility and consumption/saving as a result of improved child health and survival	Productivity
		Female labour participation
		Household investment
		Child dependency ratio
D2. Public sector budget impact	Change to an individual’s net transfers to the national budget over his/her lifetime	Return on investment
		Net present value of investment
D3. Short-term macroeconomic impact	Changes to national income or production as a result of short-term exogeneous shocks to the economy	Change in GDP (per capita)
		Change in sectoral output
D4. Long-term macroeconomic impact	Changes to national income or production as a result of long-term changes to drivers such as labour supply and foreign direct investment	Change in GDP (per capita)

^a^Most cost-effectiveness evaluations focus on maximising individual preference-based measures of health. Capabilities refer to the ability of individuals to function in particular ways, and offer an alternative way to assess the value of health-altering interventions [46]

DALY, Disability-adjusted life year; GDP, Gross domestic product; PAHO, Pan American Health Organization; QALY, Quality-adjusted life-year

### Data collection

A standardized electronic form for data collection and evaluation of the strength of evidence for each pathway was developed based on a simplified form of the Cochrane Systematic Review methodology [18]. The form was pilot tested using three randomly-selected studies to be reviewed, and modified further. Grading of Recommendations Assessment, Development and Evaluation (GRADE) criteria were then used to determine the quality of evidence behind each pathway. Each paper identified was reviewed, graded for evidence quality, and associated with a pathway in the overall framework by two independent reviewers (two of JA, NS, MEP and SS). Conflicting results in terms of evidence grade or associated pathway were flagged and resolved by consensus following discussion, or if remaining unresolved, by a senior reviewer (JY).

Randomized controlled trials were given a default quality rating of ‘high’. They were downgraded by one level for any of the following that apply and by two levels for any factor regarded as severe, up to a maximum of three:

- Limitations in the design and implementation of available studies suggesting high likelihood of bias
- Indirectness of evidence (in relation to population, intervention, comparator group, or surrogate outcomes – including hypothetical vaccines or hypothetical measures of demand in willingness to pay studies)
- Unexplained heterogeneity or inconsistency of results (including problems with subgroup analyses)
- Imprecision of results (no statistical significance at *P* <0.1)
- High probability of publication bias (including industry influence from funding or author conflict of interest, observational studies with small sample size, use of data collected automatically or from registries, or use of data collected for previous studies)

Observational studies that were methodologically sound with no obvious bias were regarded as providing ‘high’ quality evidence for the existence of pathways being evaluated if they were able to demonstrate very large effects, ‘moderate’ quality evidence if the effect sizes were large, and ‘low’ quality evidence if there were small or no effects. Observational studies that were not methodologically sound were by default considered be ‘low’ quality evidence, regardless of effect size.

Most studies reviewed were based around models rather than traditional epidemiological designs involving primary data collection. There are no GRADE criteria for quality assessment of modelling studies. In order to account for the existence of such studies as part of the evidence base, we expanded the categories of studies in GRADE to include modelling studies but kept them in a separate category from empirical studies. Table 2 below shows the grading system used.

**Table 2:** Grading of experimental, observational, and modelling studies according to the quality of evidence they provide to support causal associations between immunisation and its proposed benefits

Quality of evidence	Experimental studies	Observational studies	Modelling studies or conjecture
High	Randomised trials	Double-upgraded analytical observational studies	
Moderate	Downgraded randomized trials	Upgraded analytical observational studies	
Low	Double-downgraded randomized trials	Analytical observational studies	
Very low	Triple-downgraded randomized trials	Case series and case reports	Modelling studies or conjecture

## Results

**Fig. 1:**
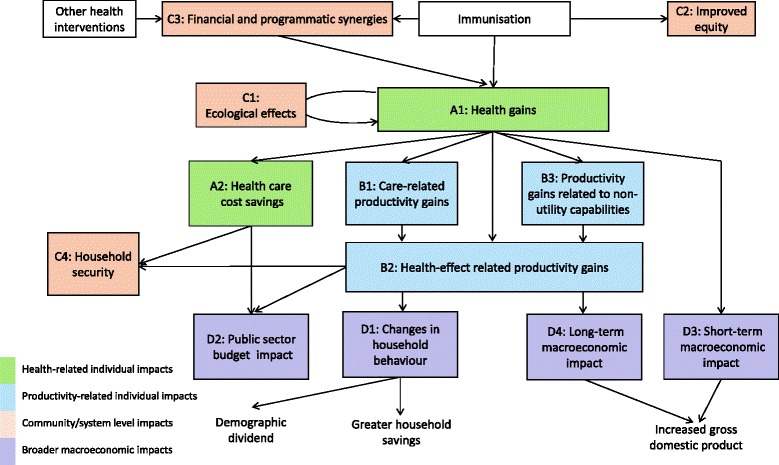
A conceptual framework for pathways to the broader economic impact of vaccines. Boxes are shaded in colours corresponding to different major categories in Table 1

Figure 1 and Table 1, respectively, provide the flow diagram and descriptions of the analytical framework arising from expert discussions about the broader economic impact of vaccines. Additional file 2 describes the relationship of our framework with previous literature in this area.

After review and screening for duplications, 93 unique journal articles from the original reviews were included in the initial study. Of these, 23 were identified as being exempt from or not appropriate for evidence quality review for reasons such as being commentaries rather than analyses. Evidence quality grades for the remaining studies are shown in Table 2; 12 additional studies were identified via expert consultation. Of these 35 studies, we identified 20 that were directly relevant to one or more of the pathways in the framework, the findings of which are summarized below. Details on the studies identified are shown in Additional file 3.

### Productivity-related benefits

#### Productivity gains due to non-utility capabilities (physical, cognitive, and education)

Evidence that immunization improves non-utility capabilities (strength of evidence = moderate experimental/observational)

No experimental studies were reviewed that directly provided evidence of the impact of immunization on the physical development of children via reduced morbidity. A single observational study by Bloom et al. [19], found no impact on physical development of adolescent children in the Philippines from the six recommended childhood vaccines. Moreover, the evidence quality itself was low, limited by small final sample size and results that are potentially inconsistent with other results on cognition from the same study.

One randomised trial of moderate quality by Canning et al. [20], provided direct evidence of the impact of maternal tetanus immunization in Matlab, Bangladesh, on the educational outcomes of children, but found that the effect was confined to only a subset of the study population. Evidence from observational studies was not strong, yet remains suggestive. Four studies point to the same overall conclusion, but all were rated low due to lack of direct causal evidence or inconsistency in the results. Two studies were based in the same study population in Bangladesh as Canning et al. [20]. Driessen et al. [21] found that measles vaccination is positively related to future school enrolment. Barham et al. [22] also found a significant relationship between cognition and exposure to a combined package of maternal and child health interventions, including, but not restricted to, routine childhood immunization. In India, Kumar [23] estimated mixed and potentially inconsistent effects on education from the Expanded Programme on Immunization. Bloom et al. [19] found a significant impact on the cognitive development of adolescent children in the Philippines (subject to the caveat above).

Evidence that immunization produces productivity gains via improvements in non-utility capabilities (strength of evidence = modelling)

By improving human capital in the form of increased physical, cognitive, and educational skills of children, immunization may have an impact on future workforce productivity and hence labour income of households as well as overall economic growth. In one modelling study, Bloom et al. [24] proposed a methodology to model these effects, but no experimental or observational studies were found with long enough follow-up to measure such effects.

### Community or health systems externalities

#### Equity (strength of evidence = moderate experimental/observational, and modelling)

In Bangladesh, Bishai et al. [25] found that the poverty-related gradient in under-five mortality is significantly reduced by measles vaccination, improving health equity directly. As previously cited, Canning et al. [9] found that maternal tetanus immunization in Bangladesh improves the educational outcomes of children in lower-income groups, which could potentially result in lower future income inequality (although none of the reviewed studies investigated income-related outcomes).

Two recent modelling studies also addressed the impact of rotavirus immunization programmes on equity. Rheingans et al. [26] modelled the distribution of potential rotavirus vaccine coverage in 25 Gavi countries by wealth quintile by extrapolating DPT2 coverage in Demographic and Health Surveys and found that, while the greatest potential benefit of rotavirus vaccination in 25 Gavi countries was in the poorest quintiles, existing rates of vaccination coverage are highly skewed towards the richest quintiles. Therefore, programmes that add new vaccines to existing systems without mechanisms to ensure equity in uptake may actually exacerbate rather than reduce existing inequity. Verguet et al. [27] modelled programme impact and consequences of rotavirus vaccination in India across different wealth strata, showing that the health and financial protection benefits of rotavirus would accrue mainly to the poor.

#### Financial and programmatic synergies and sustainability (strength of evidence = modelling)

No observational or experimental studies were found that provided direct evidence of interaction effects between immunization and other health interventions on economic outcomes. However, two modelling studies have explored the interaction between immunization and other health interventions from a cost-effectiveness perspective, showing mixed results. Jeuland et al. [28] examined combinations of new sanitation technologies and cholera vaccination and found that the incremental impact of cholera vaccination is reduced depending on the extent of a pre-existing improved water supply (although not vice versa). Tan-Torres Edejer et al. [29] examined measles immunization in South East Asia and Africa, both singly and as part of a package of nine potential interventions for child health, and found that combinations of interventions offer additive or near-additive gains (suggesting few interactions).

Several observational studies [30–32] used contingent valuation or willingness to pay methods to document substantial positive household demand for various vaccines, including HIV, dengue, and oral cholera, suggesting that households would experience private benefits from their introduction. However, these studies are limited because the vaccines examined were hypothetical only. In some cases, it is not clear that *ex ante* valuations of vaccines that did not yet exist would match *ex post* valuations in a future context. Further, while they provide evidence that significant private economic benefits exist to households, it is not possible to distinguish among the various pathways that may contribute to those benefits.

#### Household financial protection (strength of evidence = modelling)

Two mechanisms have been proposed by which immunization may reduce household financial vulnerability: firstly, through overall changes in labour earnings or savings behaviour, and secondly by reducing the risk from specific health shocks. None of the reviewed studies provided direct empirical evidence for either of these pathways. However, Verguet et al. [27] has proposed a method of ‘extended cost-effectiveness analysis’ that explicitly measures the value of the financial benefits of vaccination that accrue from risk mitigation, and applied it to a model of rotavirus vaccination in India and Ethiopia.

### Broader economic indicators

#### Changes to household behaviour (strength of evidence = conjecture)

If labour income rises, all else equal, we expect that households will be able to increase consumption, savings, and investment in human capital (such as education). Even in the absence of evidence for longer-term increases in labour income due to improved human capital, we would expect changes in immediate household consumption, savings, and investment because of increased disposable income as a result of reduced health expenditure and lost wages. However, no experimental, observational, or modelling studies were found that directly examine the effect of immunization on changes to household consumption, saving, or investment behaviour.

As the risk of childhood mortality and morbidity decreases and survival rates increase, households may also be expected to produce fewer children and invest more in their health [33]. However, no experimental, observational, or modelling studies were found that directly examine the impact of immunization on fertility decisions, and their consequent impact on households.

#### Public sector budget impact (strength of evidence = modelling)

One modelling study [34] considered the impact of the fiscal space created by the benefits of immunization by projecting lifetime net tax changes as a result of vaccination using an accounting model. However, no study provided direct primary evidence that immunization would yield such an impact.

#### Short-term macroeconomic impact (via mitigation of shocks) (strength of evidence = modelling)

No empirical studies were found that estimated the effects of vaccination in mitigating the short-term macroeconomic consequences of an infectious disease outbreak. However, several modelling studies have examined the impact of vaccination (alongside other policy responses) in the context of an influenza pandemic. Using a computable general equilibrium model, Smith et al. [35] estimated the impact of vaccination during an influenza pandemic in the United Kingdom, using either a pre-pandemic or a matched vaccine. They found that both strategies can substantially reduce the potential macroeconomic impact of a pandemic regardless of disease severity. Similarly, Keogh-Brown e al. [36] used a multi-sector single country computable general equilibrium model to evaluate the impact of antiviral, vaccination, and combined strategies in the United Kingdom, France, Belgium, and the Netherlands. They found that both vaccine-only and combined strategies would be cost-saving in either a mild or severe pandemic.

#### Long-term macro-economic impact (via changes in population health, labour force and productivity) (strength of evidence = none, but weak observational from non-vaccine fields)

We found no studies of any kind directly examining the impact that vaccination may have on long-term macro-economic indicators such as national income, growth, or foreign direct investment. However, outside the field of vaccination, there were several studies examining the relationship between improved health in general and such indicators. In a longitudinal cross-country study, Alsan et al. [37] found a positive association between population health (in terms of life expectancy) and foreign direct investment. In addition, four modelling or case studies [24, 38–40] discuss or illustrate how population or labour force changes may affect national income and growth. In other work, Soares [41] proposed a model in which changes in child mortality and life expectancy stimulate drops in fertility and rises in educational attainment, hence driving economic growth.

## Discussion

We have developed a conceptual framework of the pathways between vaccines and their proposed benefits, and assessed the strength of evidence behind each pathway. Our framework builds on previous lists of benefits [7, 10, 11], but in addition delineates potential causal relationships and outcome measures that could be used to capture each kind of benefit, and assesses the existing strength of evidence behind each proposed benefit.

We obtained mixed results from this assessment of the validity and strength of evidence behind conceptual pathways linking immunization programmes with their proposed benefits. There is indisputable evidence that vaccines bring ‘narrow’ benefits related to health outcomes, health care cost savings, and protection against productivity losses directly related to the illness episode at the level of individual vaccinees and at the community level via herd protection. There is also some limited experimental or at least observational evidence that vaccines bring wider benefits at the household level in the form of improved non-utility capabilities and equity in the distribution of health gains by wealth quintile (although one modelling study cautioned that vaccination could also widen rather than narrow inequities in health distribution). However, there is only modelling evidence to support extrapolating these benefits to meso-level household economic behaviour (in terms of demand for vaccines, consumption, savings, and investment), as well as macro-level economic indicators (such as national income, growth, and foreign investment) that might logically follow.

To incorporate modelling evidence, we expanded the traditional GRADE criteria to include a category for such studies. There was a gradient in the robustness of evidence from the modelling studies we reviewed due to variation both in the quality of input data (ranging from experimental data to observational studies or administrative records to basing assumptions purely on conjecture that human behaviour should follow particular rules) and the way the data were handled (such as the extent to which associations in non-experimental designs were assumed to imply causality, and data were extrapolated to new settings or situations). However, we did not attempt to assess the quality of modelling evidence. Although there do exist some frameworks for assessing the quality of mathematical models in health [42–44], none of these were found to be suitable for this exercise. We note that the traditional use of modelling studies is not to provide evidence for a causal relationship, but instead to assume certain previously established causal relationships in order to predict future outcomes or explore the consequences of interventions.

Our objective was to conduct a rapid review of the literature in order to validate a conceptual framework that evolved out of a consultative process. Since several systematic and narrative reviews in the area had previously been carried out, we opted to conduct a review of existing reviews with additional expert consultation, rather than a *de novo* systematic review. Previous reviews also found that there were few studies examining many of the potential broader economic benefits of vaccination [9–11]. The present study adds to the literature by aggregating the evidence from several published and unpublished reviews using different search criteria, categorising the studies based on a conceptual framework that includes causal pathways between immunization and broader outcomes, distinguishing between empirical and modelling studies, as well as explicitly grading the quality of the empirical studies.

One limitation is that we focused on the vaccine-specific literature only, and did not explore the wider literature on the relationship between health in general (or other interventions that improve health) and broader economic benefits. Since vaccines improve health, it may be reasonable to assume that the downstream relationship between health and its economic benefits also apply to vaccines. Herein, we have taken a conservative approach by ignoring any evidence relating to pathways in which immunization programmes were not the ultimate cause.

Conversely, vaccines are only one component of health systems that contribute to economic benefits. For instance, recent modelling work found that vaccines only avert around 27 % of childhood diarrhoea and pneumonia deaths, so remaining mortality reductions come from other interventions such as nutrition, case management, and hygiene [45]. The benefits of vaccines themselves depend on favourable epidemiological, environmental, socioeconomic, and health systems factors that may require investment in other interventions in health and other sectors. Hence, it is important that the attention on the broader economic benefits of vaccination does not come at the expense other public health measures. Ideally, evaluations of vaccination should be considered alongside these measures rather than individually.

Another limitation is that our framework does not consider the special benefits of maternal vaccination, including improved maternal and child health outcomes following vaccines such as influenza. Further, our framework has focused on the broader economic benefits of vaccination. However, the broader economic costs of vaccination are also of increasing interest to decision making. These include costs related to addressing adverse effects following immunisation and vaccine hesitancy at a societal level.

The absence of evidence from interventional studies to support many of the proposed pathways does not imply evidence for the absence of such relationships. Many vaccines have proven health and economic benefits and are being routinely used in most countries, so experimental designs that deny vaccines to a proportion of participants may be considered unethical. Furthermore, associations between interventions and long-term economic behaviour at the household or macroeconomic level are expensive and time-consuming to investigate experimentally. Conditions for well-controlled ‘natural’ experiments are also rare. Hence, the use of designs that would be considered robust in the medical literature may be less common in the economics literature. Similarly, assumptions about human behaviour (such as rationality) may be well-accepted in in the economics literature without the need for empirical justification, but this is not the case in the medical literature. This may explain the decrease in availability of experimental or even observational evidence as we move from health-related outcomes to economic outcomes at either the household or the macro-economic level.

## Conclusions

The present study highlights the importance of collecting data about such behaviour wherever possible in order to strengthen the evidence base behind the potential broader economic impacts of vaccination. For instance, some of the outcomes for which we did not find experimental evidence (such as household choices around consumption and savings) could be routinely incorporated into clinical effectiveness trials of vaccines or into standard demographic and health surveys, should suitable data collection instruments be developed. In the interim, innovative analyses of historical observational datasets may provide evidence that would be considered more robust than models whose assumptions are not driven by data. There is a need to design the requisite methods and initiate the relevant studies as soon as possible given the increasing importance of broader economic indicators of vaccine impact in informing investment decisions about vaccines.

## Additional files

Additional file 1:
**List of participants in expert consultations on the broader economic impact of vaccination and immunisation programmes convened by the World Health Organization.** Participants at the meetings in Toronto (13–14 July 2011), Geneva (28–29 June 2012), Sydney (10–11 July 2013), and Bangkok (24–25 November 2014). (DOCX 20 kb) Additional file 2:
**Relationship of the framework with previous lists of categories of broader economic impact of vaccination and immunisation programmes.** List of categories of broader economic impact included in each list, and flow diagram showing the development of the current framework. (DOCX 159 kb) Additional file 3:
**Details of the studies found in the systematic review.** I. Evidence quality rating of potentially admissible studies. II. Studies on willingness-to-pay, cost-effectiveness, and direct health outcomes. III. Categorisation of relevant studies into potential benefits of vaccination. (DOCX 52 kb)

## Abbreviation

GRADE: Grading of Recommendations Assessment, Development and Evaluation
