# Supplementary material for: The broader economic impact of vaccination: reviewing and appraising the strength of evidence
Source: BMC Med. 2015 Sep 3;13:209. doi: 10.1186/s12916-015-0446-9 (PMC4558933; doi:10.1186/s12916-015-0446-9)
Supplement: Additional file 1: — List of participants in expert consultations on the broader economic impact of vaccination and immunisation programmes convened by the World Health Organization. Participants at the meetings in Toronto (13–14 July 2011), Geneva (28–29 June 2012), Sydney (10–11 July 2013), and Bangkok (24–25 November 2014). (DOCX 20 kb) [file 12916_2015_446_MOESM1_ESM.docx]

**Additional file 1. List of participants in expert consultations on the broader economic impact of vaccination and immunisation programmes convened by the World Health Organization**

**Toronto (13-14 July 2011)**

Arnab Acharya, London School of Hygiene and Tropical Medicine

Til Baernighausen, Harvard University

Jose Ricardo de Mello Brandao, Centre for Global Health Research, University of Toronto.

Anaïs Colombini, Agence de Médecine Préventive

Dagna Constenla, John Hopkins University

Rohan Deogaonkar, University of Birmingham, UK

Silvia Evers, Maastricht University

Raymond Hutubessy, World Health Organization

Mark Jit, Health Protection Agency

Mira Johri, University of Montreal

Ann Levin, Independent consultant

Arindam Nandi, Center for Disease Dynamics, Economics & Policy

Jennifer O’Brien, Harvard University

Maarten Postma, University of Groningen

Inge van der Putten, Maastricht University

Baudouin Standaert, GlaxoSmithKline Biologicals

Aparnaa Somanathan, World Bank

Stephane Verguet, University of Washington

Damian Walker, Bill and Melinda Gates Foundation

**Geneva (28-29 June 2012)**

Til Baernighausen, Harvard University

Mark Connolly, Global Market Access Solutions Sarl

Philippe Beutels, University of Antwerp

John Edmunds, London School of Hygiene and Tropical Medicine

Peter Hansen, GAVI Alliance

Raymond Hutubessy, World Health Organization

Mark Jit, Health Protection Agency

Milloud Kaddar, World Health Organization

Nikos Kotsopoulos, Global Market Access Solutions Sarl

Andrew Steer, University of Melbourne

Inge van der Putten, World Health Organization

Stephane Vergeut, University of Washington

Jonathan Weiss, Independent consultant

**Sydney (10-11 July 2013)**

Ariel Benyishay, University of New South Wales

Jean-Bernard Le Gargasson, Agence de Médecine Préventive, Ferney-Voltaire, France

Logan Brenzel, World Bank

Marc Brisson, Laval University

Nathalie Carvalho, University of Melbourne

Mark Connolly, Global Market Access Solutions Sarl

Ulla Griffiths, London School of Hygiene and Tropical Medicine

Mark Jit, London School of Hygiene and Tropical Medicine

Miloud Kaddar, World Health Organization

Ann Levin, Consultant, Bethesda, USA

Anthony Newall, University of New South Wales

Sachiko Ozawa, John Hopkins University

Allison Portnoy, John Hopkins University

Stephen Resch, Harvard University

Josephine Reyes, University of New South Wales

Stephane Verguet, University of Washington

David Wilson, University of New South Wales

Virginia Wiseman, University of New South Wales

**Bangkok (24-25 November 2014)**

Taiwo Abimbola, Centers For Disease Control and Prevention,

Alex Adjagba, Agence de Médecine Préventive

Rashmi Arora, Indian Council of Medical Research

Phing Tze Chai, University of Malaya

Nathorn Chaiyakunapruk, Monash University Sunway Campus (Malaysia)

Samantha Clark, John Hopkins University

Lou Garrison, University of Washington

Mohan Gupte, Indian Council of Medical Research

Raymond Hutubessy, World Health Organization, Geneva

Mark Jit, London School of Hygiene and Tropical Medicine

Jahangir Khan, International Centre for Diarrhoeal Disease Research (ICDDR,B)

Soewarta Kosen, Institute of Health Research & Development (Indonesia)

Jeremy Lauer, World Health Organization

Jean-Bernard Le Gargasson, Agence de Médecine Préventive

Tharani Loganathan, University of Malaya

Vital Mogasale, International Vaccine Institute

Anthony Nelson, Chinese University of Hong Kong

Anthony Newall, University of New South Wales

Chiu Wan Ng, University of Malaya

Lucas Otieno, KEMRI/Walter Reed Army Institute of Research

Sachiko Ozawa, John Hopkins University

Allison Portnoy, John Hopkins University

Naiyana Praditsitthikorn, Health Intervention and Technology Assessment Program (Thailand)

Richard Rheingans, University of Florida

Arthorn Riewpaiboon, Mahidol University

Montarat Thavorncharoensap, Mahidol University

Nguyen Tran Hien, National Institute of Hygiene and Epidemiology (Viet Nam)

Inge van der Putten, Maastricht University

Kaya Verbooy, National University of Singapore, Singapore

Stephane Verguet, University of Washington

Suwit Wibulpolprasaert, Ministry of Public Health (Thailand)

Joanne Yoong, National University of Singapore, Singapore
