# Supplementary material for: The broader economic impact of vaccination: reviewing and appraising the strength of evidence
Source: BMC Med. 2015 Sep 3;13:209. doi: 10.1186/s12916-015-0446-9 (PMC4558933; doi:10.1186/s12916-015-0446-9)
Supplement: Additional file 2: — Relationship of the framework with previous lists of categories of broader economic impact of vaccination and immunisation programmes. List of categories of broader economic impact included in each list, and flow diagram showing the development of the current framework. (DOCX 159 kb) [file 12916_2015_446_MOESM2_ESM.docx]

**Additional file 3. Relationship of the framework with previous lists of categories of broader economic impact of vaccination and immunisation programmes.**

A list of “broader” benefits of vaccination that are often overlooked in traditional economic evaluations was first proposed in Bärnighausen et al. (2008) [1] and expanded in Bärnighausen et al. (2014) [2]. The original (2008) list was the basis of two independent systematic reviews of economic evaluations of vaccination (Ozawa et al., 2012 [3]; Deogaonkar et al., 2012 [4]), both of which expanded the list of benefits after finding additional categories not included in the original lists by Bärnighausen and colleagues. The list of categories proposed by Deogaonkar and colleagues was expanded following the Geneva (2012) WHO consultation. During the Sydney (2013) WHO consultation this was further expanded, including considering the list of categories proposed by Ozawa and colleagues.

The figure below shows the historical relationship and development of these frameworks.

The following table shows the categories of benefit that are included in each of the published lists of such benefits.

| **Category of benefit** | **Bärnighausen et al. (2008) [1]** | **Bärnighausen et al. (2014) [2]** | **Ozawa et al. (2012) [3]** | **Deogaonkar et al. (2012) [4]** | **This paper** |
| --- | --- | --- | --- | --- | --- |
| A1. Health gains | √ | √ | √ | √ | √ |
| A2. Health care cost savings | √ | √ | √ | √ | √ |
| B1. Productivity gains related to care | √ | √ | √ | √ | √ |
| B2. Productivity gains related to health effects | Outcome-related productivity gains | Outcome-related productivity gains | Outcome-related productivity gains | √ | √ |
| B3.Productivity gains related to non-utility capabilities |  |  |  | √ | √ |
| C1. Ecological effects | Community externalities | Community health externalities |  |  | √ |
| C2. Equity |  |  |  | √ | √ |
| C3. Financial and programmatic synergies and sustainability |  |  |  | √ | √ |
| C4. Household security |  |  |  |  | √ |
| D1. Changes to household behaviour | Behaviour-related productivity gains | Behaviour-related productivity gains | Behaviour-related productivity gains | Productivity gains related to household behaviour |  |
| D2. Public sector budget impact |  |  |  |  | √ |
| D3. Short-term macroeconomic impact |  | Community economic externalities | Outbreak cost savings | Macroeconomic impact | √ |
| D4. Long-term macroeconomic impact |  |  |  |  | √ |
| Risk reduction gains |  | √ |  |  |  |
| Willingness to pay & value of statistical life |  |  | √ |  |  |

Our framework encompasses all the benefit categories described in previous lists apart from “risk reduction gains” and “willingness to pay & value of statistical life”. “Risk reduction gains” are “gains in welfare because uncertainty in future outcomes is reduced” [2]. “Willingness to pay & value of statistical life” are “individuals or society’s economic valuation of the long-term benefits from vaccination, including productivity gains and benefits of averting pain and suffering from vaccine-preventable diseases” [3]. These are arguably methods of presenting the benefits of vaccination (including some not captured in many traditional cost-effectiveness analyses), usually within the framework of uncertainty analysis (for risk reduction gains) and cost-benefit analysis (for willingness to pay & value of statistical life).

**References**

1. Bärnighausen T, Bloom DE, Canning D, O’Brien J: **Accounting for the full benefits of childhood vaccination in South Africa.** *S Afr Med J* 2008, **98**:842, 844–6.

2. Bärnighausen T, Bloom DE, Cafiero-Fonseca ET, O’Brien JC: **Valuing vaccination.** *Proc Natl Acad Sci U S A* 2014, **111**:12313–9.

3. Ozawa S, Mirelman A, Stack ML, Walker DG, Levine OS: **Cost-effectiveness and economic benefits of vaccines in low- and middle-income countries: a systematic review.** *Vaccine* 2012, **31**:96–108.

4. Deogaonkar R, Hutubessy R, van der Putten I, Evers S, Jit M: **Systematic review of studies evaluating the broader economic impact of vaccination in low and middle income countries.** *BMC Public Health* 2012, **12**:878.
