# Supplementary material for: The broader economic impact of vaccination: reviewing and appraising the strength of evidence
Source: BMC Med. 2015 Sep 3;13:209. doi: 10.1186/s12916-015-0446-9 (PMC4558933; doi:10.1186/s12916-015-0446-9)
Supplement: Additional file 3: — Details of the studies found in the systematic review. I. Evidence quality rating of potentially admissible studies. II. Studies on willingness-to-pay, cost-effectiveness, and direct health outcomes. III. Categorisation of relevant studies into potential benefits of vaccination. (DOCX 52 kb) [file 12916_2015_446_MOESM3_ESM.docx]

**Additional file 2. Details of the studies found in the systematic review.**

**Contents**

[I. Evidence quality rating of potentially admissible studies 1](#_Toc417940774)

[II. Studies on willingness-to-pay, cost-effectiveness and direct health outcomes 8](#_Toc417940775)

[III. Categorisation of relevant studies into potential benefits of vaccination 13](#_Toc417940776)

# I. Evidence quality rating of potentially admissible studies

| Citation | Evidence Score |
| --- | --- |
| Laxminarayan, R. Fighting antibiotic resistance: Can economic incentives play a role? Resources 2001, 143: 9-12. | NA |
| Knodel, J. and E. Vandewalle. Lessons from the Past - Policy Implications of Historical Fertility Studies. Population and Development Review 1979; 5:217-245. | NA |
| Dahan M, Tsiddon D. Demographic transition, income distribution, and economic growth. Journal of Economic Growth 1998, 3:29–52. | NA |
| Bloom DE, Canning D, Weston M. The value of vaccination. World Economics 2005, 6:15–39. | NA |
| Beutels P, Edmunds WJ, Smith RD. Partially wrong? Partial equilibrium and the health economic analysis of public health emergencies of international concern. Health Econ 2008, 17:1317–1322. | NA |
| Connolly MP, Postma MJ. Healthcare as an investment: Implications for an era of ageing populations. J Med Mark 2010;10(1):5-14. | NA |
| Alsan M, Canning D, Bloom D E. The effect of population health on foreign direct investments. NBER Working Paper Series 2004; Working Paper 10596. | NA |
| Bloom DE, Canning D, Weston M. The value of vaccination. World Economics 2005, 6:15–39. | NA |
| Murray CJL et al. (2012). Disability-adjusted life years (DALYs) for 291 diseases and injuries in 21 regions, 1990—2010: a systematic analysis for the Global Burden of Disease Study 2010. The Lancet, 380 (9859): 2197-2223 | NA |
| Murray CJ, Lopez AD. The Global Burden of Disease: a comprehensive assessment of mortality and disability from diseases, injuries and risk factors in 1990 and projected to 2020. Cambridge, MA, Harvard School of Public Health, (Global Burden of Disease and Injury Series, vol. I) | NA |
| Koopmanschap MA, Rutten FF, van Ineveld BM, van Roijen L. The friction cost method for measuring indirect costs of disease. J Health Econ. 1995 Jun, 14(2):171-89. | NA |
| Barrett S. Eradication versus control: the economics of global infection disease policy. Bulletin of the World Health Organization 2004; 82: 683-688. | NA |
| Connolly M,  Constenla D. Assessing economic benefits for government and society attributed to malaria investment strategies: An exploratory analysis based on malaria vaccination. In Proceedings of The Multilateral Initiative on Malaria 2009: www.gmasoln.com/downloads/Malaria.pdf. | NA |
| Drummond M, Chevat C, LothgrenM. Do we fully understand the economic value of vaccines? Vaccine 2007; 25: 5945–5957. | NA |
| Bärnighausen T, Bloom DE, Canning D, Friedman A, Levine O, O’Brien J, Privor-Dumm J, Walker D (2011). Rethinking the benefits and costs of childhood vaccination: the example of the Haemophilus influenza type b vaccine. Vaccine, 29(13): 2371-2380. | NA |
| Goldie SJ, O’Shea M, Diaz M, Kim S-Y. Benefits, cost requirements and cost-effectiveness of the HPV16,18 vaccine for cervical cancer prevention in developing countries: policy implications. Reprod Health Matters 2008, 16(32):86–96. | NA |
| World Health Organisation. The Global Burden of Disease: 2004 Update. World Health Organisation; Geneva Switzerland; 2008. | NA |
| Lazano R et al. (2012).Global and regional mortality from 235 causes of death for 20 age groups in 1990 and 2010: a systematic analysis for the Global Burden of Disease Study 2010.The Lancet, 380(9859): 2095-2128. | NA |
| Global Burden of Disease Study 2010. Global Burden of Disease Study 2010 Results by Cause 1990-2010. Seattle, United States: Institute for Health Metrics and Evaluation (IHME), 2012. | NA |
| Simmerman JM, Lertiendumrong J, Dowell SF, Uyeki T, Olsen SJ, Chittaganpitch M, et al. The cost of influenza in Thailand. Vaccine 2006, 240:4417–26. | NA |
| Berndt ER, Glennerster R, Kremer MR, Lee J, Levine R, Weizsäcker G, et al. Advance market commitments for vaccines against neglected diseases: estimating costs and effectiveness. Health Econ 2007, 16(5):491–511. | NA |
| Vespa G, Constenla DO, Pepe C, Safadi MA, Berezin E, de Moraes JC, et al. Estimating the cost-effectiveness of pneumococcal conjugate vaccination in Brazil. Rev Panam Salud Pública 2009, 26(6):518–28. | 4 |
| Goldie SJ, O’Shea M, Campos NG, Diaz M, Sweet S, Kim S-Y. Health and economic outcomes of HPV 16,18 vaccination in 72 GAVI-eligible countries. Vaccine 2008, 26(32):4080–93. | 4 |
| Kim S-Y, Salomon J, Goldie S. Economic evaluation of hepatitis B vaccination in low-income countries: using cost-effectiveness affordability curves. Bull World Health Organ 2007, 85(11):833–42. | 4 |
| Lopez E,  Debbag R, Coudeville L, Baron-Papillon F, Armoni J. The cost-effectivness of universal vaccination of children against hepatitis A in Argentina: results of a dynamic health-economic analysis. J Gastroenterology 2007, 42:152–160. | 4 |
| Atherly D, Dreibelbis R, Parashar Umesh D, Levin C, Wecker J, Rheingans Richard D. Rotavirus vaccination: cost effectiveness and impact on child mortality in developing countries. J Infect Dis 2009, 200(s1):S28–38. | 4 |
| Bishai D, Johns B, Lefevre A, Nair D. Cost effectiveness of measles eradication. World Health Organization 2010. | 4 |
| Clark AD, Walker DG, Mosqueira NR, Penny ME, Lanata CF, Fox-Rushby J, et al. Cost-effectiveness of rotavirus vaccination in Peru. J Infect Dis 2009, 200(Suppl. 1):S114–24. | 4 |
| Constenla D, Velázquez FR, Rheingans RD, Antil L, Cervantes Y. Economic impact of a rotavirus vaccination program in Mexico. Rev Panam Salud Pública 2009, 25(6):481–90. | 4 |
| Tate JE, Rheingans RD, O’Reilly CE, Obonyo B, Burton DC, Tornheim JA, et al. Rotavirus disease burden and impact and cost-effectiveness of a rotavirus vaccination program in Kenya. J Infect Dis 2009, 200(Suppl. 1): S76–84. | 4 |
| Ding D, Kilgore P, Clemens J, Wei L, Zhi-Yi X. Cost-effectiveness of routine immunization to control Japanese encephalitis in Shanghai, China. Bull World Health Organ 2003, 81(5):334–42. | 4 |
| Flem ET, Latipov R, Nurmatov ZS, Xue Y, Kasymbekova KT, Rheingans RD. Costs of diarrheal disease and the cost-effectiveness of a rotavirus vaccination program in Kyrgyzstan. J Infect Dis 2009, 200(November (Suppl. 1)):S195–202. | 4 |
| Jeuland M, Lucas M, Clemens J, Whittington D. A Cost–Benefit Analysis of Cholera Vaccination Programs in Beira, Mozambique. World Bank Econ Rev 2009, 23:235–267. | 4 |
| Constenla DO, Linhares AC, Rheingans RD, Antil LR, Waldman EA, da Silva LJ. Economic impact of a rotavirus vaccine in Brazil. J Health Popul Nutr 2008, 26(4):388–96. | 4 |
| Edejer TTT. Cost effectiveness analysis of strategies for child health in developing countries. Br Med J 2005, 331(7526):1177–80. | 4 |
| Touch S, Suraratdecha C, Samnang C, Heng S, Gazley L, Huch C, et al. A cost-effectiveness analysis of Japanese encephalitis vaccine in Cambodia. Vaccine 2010, 28(29):4593–9. | 4 |
| Duintjer Tebbens RJ, Pallansch MA, Cochi SL, Wassilak SGF, Linkins J, Sutter RW, et al. Economic analysis of the global polio eradication initiative. Vaccine 2010, 29(2):43–334. | 4 |
| Stack ML,  Ozawa S, Bishai DM, Mirelman A, Tam Y, Niessen L, Walker DG, Levine OS. Estimated economic benefits during the ‘Decade of Vaccines’ include treatment savings, gains in labor productivity. Health Aff 2011, 30:1021–1028. | 4 |
| Ozawa S, Stack ML, Bishai DM, Mirelman A, Friberg IK, Niessen L, Walker DG, Levine OS. During the ‘Decade of Vaccines’ the lives of 6.4 million children valued at $231 billion could be saved. Health Aff 2011, 30:1010–1020. | 4 |
| Bishai D, Koenig M, Khan MA. Measles vaccination improves the equity of health outcomes: evidence from Bangladesh. Health Econ 2003, 12:415–419. | 4 |
| Meij JJ, de Craen AJ, Agana J, Plug D, Westendorp RG. Low-cost interventions accelerate epidemiological transition in Upper East Ghana. Trans R Soc Trop Med Hyg 2009;103(February (2)):173–8. | 4 |
| Ortega O, El-Sayed N, Sanders JW, Abd-Rabou Z, Antil L, Bresee J, et al. Cost–benefit analysis of a rotavirus immunization program in the Arab Republic of Egypt. J Infect Dis 2009, 200(November (Suppl. 1)):S92–8. | 3 |
| Jeuland M, Whittington D. Cost-benefit comparisons of investments in improved water supply and cholera vaccination programs. Vaccine 2009, 27:3109–3120. | 3 |
| Niessen L, ten Hove A, Hilderink H, Weber M, Mulholland K, Ezzati M. Comparative impact assessment of child pneumonia interventions. Bull World Health Organ 2009, 87:472–480. | 3 |
| Tediosi F, Hutton G, Maire N, Smith TA, Ross A, Tanner M. Predicting the cost-effectiveness of introducing a pre-erythrocytic malaria vaccine into the expanded program on immunization in Tanzania. Am J Trop Med Hyg 2006, 75(2 Suppl.):131–43. | 3 |
| Isakbaeva ET , Musabaev E, Antil L, Rheingans R, Juraev R, Glass RI, et al. Rotavirus disease in Uzbekistan: cost-effectiveness of a new vaccine. Vaccine 2007, 25(January (2)):373–80. | 3 |
| Kim S-Y, Goldie SJ, Salomon JA. Cost-effectiveness of rotavirus vaccination in Vietnam. BMC Public Health 2009, 9(1):29. | 3 |
| Platonov AE, Griffiths UK, Voeykova MV, Platonova OV, Shakhanina IL, Chistyakova GG, et al. Economic evaluation of Haemophilus influenzae type b vaccination in Moscow, Russian Federation. Vaccine 2006, 24(March (13)):2367–76. | 3 |
| Griffiths UK, Hutton G, Das Dores Pascoal E. The cost-effectiveness of introducing hepatitis B vaccine into infant immunization services in Mozambique. Health Policy Plan 2005, 20(January (1)):50–9. | 3 |
| Khan MM. Economics of polio vaccination in the post-eradication era: should OPV-using countries adopt IPV? Vaccine 2008, 26(16):2034–40. | 3 |
| Jeuland M, Cook J, Poulos C, Clemens J, Whittington D. Cost-effectiveness of new-generation oral cholera vaccines: a multisite analysis. Value Health 2009, 12(6):899–908. | 3 |
| Rheingans Richard D, Antil L, Dreibelbis R, Podewils Laura J, Bresee Joseph S, Parashar Umesh D. Economic costs of rotavirus gastroenteritis and cost effectiveness of vaccination in developing countries. J Infect Dis 2009, 200(s1):S16–27. | 3 |
| Prakash C. Crucial factors that influence cost-effectiveness of universal hepatitis B immunization in India. Int J Technol Assess Health Care 2003, 19(Winter (1)):28–40. | 3 |
| Berry SA, Johns B, Shih C, Berry AA, Walker DG. The cost-effectiveness of rotavirus vaccination in Malawi. J Infect Dis 2010, 202(Suppl.):S108–15. | 3 |
| Broughton EI. Economic evaluation of Haemophilus influenzae type B vaccination in Indonesia: a cost-effectiveness analysis. J Public Health 2007, 29(4):441–8. | 3 |
| Cook J, Jeuland M, Whittington D, Poulos C, Clemens J, Sur D, et al. The cost- effectiveness of typhoid Vi vaccination programs: calculations for four urban sites in four Asian countries. Vaccine 2008, 26(November (50)):6305–16. | 3 |
| Sinha A, Levine O, Knoll MD, Muhib F, Lieu TA. Cost-effectiveness of pneumococcal conjugate vaccination in the prevention of child mortality: an international economic analysis. Lancet 2007, 369(9559): 389–96. | 3 |
| Gessner BD, Sedyaningsih ER, Griffiths UK, Sutanto A, Linehan M, Mercer D, et al. Vaccine-preventable haemophilus influenza type B disease burden and cost-effectiveness of infant vaccination in Indonesia. Pediatr Infect Dis J 2008, 27(5):438–43. | 3 |
| Constenla DO. Economic impact of pneumococcal conjugate vaccination in Brazil, Chile, and Uruguay. Rev Panam Salud Pública 2008, 24(2): 101–12. | 3 |
| Griffiths UK, Botham L, Schoub BD. The cost-effectiveness of alternative polio immunization policies in South Africa. Vaccine 2006, 24(July (29–30)):5670–8. | 3 |
| Fischer TK, Anh DD, Antil L, Cat NDL, Kilgore PE, Thiem VD, et al. Health care costs of diarrheal disease and estimates of the cost-effectiveness of rotavirus vaccination in Vietnam. J Infect Dis 2005,192(10):1720–6. | 3 |
| Wilopo SA, Kilgore P, Kosen S, Soenarto Y, Aminah S, Cahyono A, et al. Economic evaluation of a routine rotavirus vaccination programme in Indonesia. Vaccine 2009,27(November (Suppl. 5)):F67–74. | 3 |
| Canning D, Razzaque A, Driessen J, Walker D G, Streatfield P K, Yunus M. The effect of maternal tetanus immunization on children’s schooling attainment in Matlab, Bangladesh: follow-up of a randomized trial. Social Science & Medicine 2011; 72: 1429-1436. | 3 |
| Ono S, Kurotaki T, Nakasone T, Honda M, Boon-Long J, Sawanpanyalert P, et al. Cost-effectiveness analysis of antiretroviral drug treatment and HIV-1 vaccination in Thailand. Jpn J Infect Dis 2006, 59:168–73. | 2 |
| Podewils LJ, Antil L, Hummelman E, Bresee J, Parashar UD, Rheingans R. Projected cost-effectiveness of rotavirus vaccination for children in Asia. J Infect Dis 2005, 192(Suppl. 1):S133–45. | 2 |
| Sinha A, Constenla D, Valencia JE, O’Loughlin R, Gomez E, de la Hoz F, et al. Cost-effectiveness of pneumococcal conjugate vaccination in Latin America and the Caribbean: a regional analysis. Rev Panam Salud Pública 2008, 24(5):304–13. | 2 |
| Chotivitayatarakorn P, Poovorawan Y. Cost-effectiveness of rotavirus vaccination as part of the national immunization program for Thai children. Southeast Asian J Trop Med Public Health 2010, 41:114–25. | 2 |
| Giglio ND, Cane AD, Micone P, Gentile A. Cost-effectiveness of the CRM-based 7- valent pneumococcal conjugated vaccine (PCV7) in Argentina. Vaccine 2010, 28:2302– 2310. | 2 |
| Akumu AO, English M, Scott JA, Griffiths UK. Economic evaluation of delivering Haemophilus influenzae type b vaccine in routine immunization services in Kenya. Bull World Health Organ 2007, 85(July (7)):511–8. | 2 |
| Kim S-Y, Lee G, Goldie SJ. Economic evaluation of pneumococcal conjugate vaccination in The Gambia. BMC Infect Dis 2010, 10:260. | 2 |
| Cook J, Jeuland M, Maskery B, Lauria D, Sur D, Clemens J, et al. Using private demand studies to calculate socially optimal vaccine subsidies in developing countries. J Policy Anal Manage 2009;28(1):6–28. | 2 |
| Parent du Châtelet I, Gessner BD, da Silva A. Comparison of cost-effectiveness of preventive and reactive mass immunization campaigns against meningococcal meningitis in West Africa: a theoretical modeling analysis. Vaccine 2001, 19(25–26):3420–31. | 2 |
| Bawah AA, Phillips JF, Adjuik M, Vaughan-Smith M, Macleod B, Binka FN. The impact of immunization on the association between poverty and child survival: Evidence from Kassena-Nankana district of northern Ghana. Scand J Public Health 2010, 38:95–103. | 2 |
| Kim D, Canh do G, Poulos C, Thoa le TK, Cook J, Hoa NT, et al. Private demand for cholera vaccines in Hue, Vietnam. Value Health 2008;11(January–February (1)):28–119. | 2 |
| Lucas ME, Jeuland M, Deen J, Lazaro N, MacMahon M, Nyamete A, et al. Private demand for cholera vaccines in Beira, Mozambique. Vaccine 2007;25(March (14)):2599–609. | 2 |
| Novaes H, Luna E, Goldbaum M, Kilsztajn S, Rossbach A, de la Rocha Carval- heiro J. The potential demand for an HIV/AIDS vaccine in Brazil. World Bank policy research working paper. The World Bank; 2002 p. 1–30. | 2 |
| Cropper M, Haile M, Lampietti J, Poulos C, Whittington D. The demand for a malaria vaccine: evidence from Ethiopia. J Dev Econ 2004;75:303–18. | 2 |
| Whittington D, Sur D, Cook J, Chatterjee S, Maskery B, Lahiri M, et al. Rethinking cholera and typhoid vaccination policies for the poor: private demand in Kolkata, India. World Dev 2009;37(2):399–409. | 2 |
| Whittington D, Matsui-Santana O, Freiberger JJ, Van Houtven G, Pattanayak S. Private demand for a HIV/AIDS vaccine: evidence from Guadalajara, Mexico. Vaccine 2002;20(June (19–20)):2585–91. | 2 |
| Rheingans RD, Constenla D, Antil L, Innis BL, Breuer T. Potential cost-effectiveness of vaccination for rotavirus gastroenteritis in eight Latin American and Caribbean countries. Rev Panam Salud Publica 2007, 21(April (4)):205–16. | 1 |
| De la Hoz F, Alvis N, Narváez J, Cediel N, Gamboa O, Velandia M. Potential epidemiological and economical impact of two rotavirus vaccines in Colombia. Vaccine 2010, 28(22):3856–64. | 1 |
| Udezi WA, Usifoh CO, Ihimekpen OO. Willingness to pay for three hypothetical malaria vaccines in Nigeria. Clin Ther 2010;32(August (8)):1533–44. | 1 |
| Palanca-Tan R. The demand for a dengue vaccine: a contingent valuation survey in Metro Manila. Vaccine 2008;26(February (7)):914–23. | 1 |
| Sauerborn R, Gbangou A, Dong H, Przyborski JM, Lanzer M. Willingness to pay for hypothetical malaria vaccines in rural Burkina Faso. Scand J Public Health 2005;33(2):146–50. | 1 |
| Whittington D, Suraratdecha C, Poulos C, Ainsworth M, Prabhu V, Tangcharoensathien V. Household demand for preventive HIV/AIDS vaccines in Thailand: do husbands’ and wives’ preferences differ? Value Health 2008;11(September–October (5)):965–74. | 1 |
| Islam Z, Maskery B, Nyamete A, Horowitz MS, Yunus M, Whittington D. Private demand for cholera vaccines in rural Matlab, Bangladesh. Health Policy 2008;85(February (2)):184–95. | 1 |
| Jeuland M, Lucas M, Clemens J, Whittington D. Estimating the private benefits of vaccination against cholera in Beira, Mozambique: a travel cost approach. J Dev Econ 2010;91:310–22. | 1 |
| Barham T, Calimeris L. Long-term effects of family planning and child health interventions on adolescent cognition: Evidence from Matlab in Bangladesh. Working paper. University of Colorado: Health and Society program; 2008. | 1 |
| Bloom D E, Canning D, Seiguer E. The effect of vaccination on children's physical and cognitive development in the Philippines. Applied Economics 2012; 44: 2777–2783. | 1 |
| Kumar S. Childhood immunization, mortality and human capital accumulation: Micro- evidence from India. Working paper. University of Houston: Harvard Centre for Population and Development Studies; 2009. http://mpra.ub.uni-muenchen.de/31806/. | 1 |
| Driessen J, Razzaque A, Walker D, Canning D. The effect of childhood measles vaccination on school enrolment in Matlab, Bangladesh. PGDA Working Paper 2011; 81 | 1 |
| Apisarnthanarak A, Puthavathana P, Kitphati R, Auewarakul P, Mundy L. Outbreaks of Influenza A among nonvaccinated healthcare workers: implications for resource-limited settings. Infect Control Hosp Epidemiol 2008, 29(8):777–80. | 1 |
| Heinzen RR, Bridges JF. Comparison of four contingent valuation methods to estimate the economic value of a pneumococcal vaccine in Bangladesh. Int J Technol Assess Health Care 2008;24(Fall (4)):481–7. | 1 |

# II. Studies on willingness-to-pay, cost-effectiveness and direct health outcomes

**Willingness-To-Pay Studies**

| Citation | Location | Vaccine |
| --- | --- | --- |
| Whittington D, Sur D, Cook J, Chatterjee S, Maskery B, Lahiri M, et al. Rethinking cholera and typhoid vaccination policies for the poor: private demand in Kolkata, India. World Dev 2009;37(2):399–409. | India | Cholera and typhoid |
| Palanca-Tan R. The demand for a dengue vaccine: a contingent valuation survey in Metro Manila. Vaccine 2008;26(February (7)):914–23. | Phillippines | Dengue |
| Novaes H, Luna E, Goldbaum M, Kilsztajn S, Rossbach A, de la Rocha Carval- heiro J. The potential demand for an HIV/AIDS vaccine in Brazil. World Bank policy research working paper. The World Bank; 2002 p. 1–30. | Brazil | HIV/AIDS |
| Whittington D, Matsui-Santana O, Freiberger JJ, Van Houtven G, Pattanayak S. Private demand for a HIV/AIDS vaccine: evidence from Guadalajara, Mexico. Vaccine 2002;20(June (19–20)):2585–91. | Mexico | HIV/AIDS |
| Whittington D, Suraratdecha C, Poulos C, Ainsworth M, Prabhu V, Tangcharoensathien V. Household demand for preventive HIV/AIDS vaccines in Thailand: do husbands’ and wives’ preferences differ? Value Health 2008;11(September–October (5)):965–74. | Thailand | HIV/AIDS |
| Sauerborn R, Gbangou A, Dong H, Przyborski JM, Lanzer M. Willingness to pay for hypothetical malaria vaccines in rural Burkina Faso. Scand J Public Health 2005;33(2):146–50. | Burkina Faso | Malaria |
| Cropper M, Haile M, Lampietti J, Poulos C, Whittington D. The demand for a malaria vaccine: evidence from Ethiopia. J Dev Econ 2004;75:303–18. | Ethiopia | Malaria |

**Studies on direct health outcomes**

| Citation | Location | Vaccine |
| --- | --- | --- |
| Duintjer Tebbens RJ, Pallansch MA, Cochi SL, Wassilak SGF, Linkins J, Sutter RW, et al. Economic analysis of the global polio eradication initiative. Vaccine 2010, 29(2):43–334. | Global | Polio |
| Stack ML,  Ozawa S, Bishai DM, Mirelman A, Tam Y, Niessen L, Walker DG, Levine OS. Estimated economic benefits during the ‘Decade of Vaccines’ include treatment savings, gains in labor productivity. Health Aff 2011, 30:1021–1028. | Global | pneumococcal and Haemophilus influenzae type b pneumonia and meningitis, rotavirus, pertussis, measles, and malaria |
| Ozawa S, Stack ML, Bishai DM, Mirelman A, Friberg IK, Niessen L, Walker DG, Levine OS. During the ‘Decade of Vaccines’ the lives of 6.4 million children valued at $231 billion could be saved. Health Aff 2011, 30:1010–1020. | Global | pneumococcal and Haemophilus influenzae type b pneumonia and meningitis, rotavirus, pertussis, measles, and malaria |
| Bishai D, Koenig M, Khan MA. Measles vaccination improves the equity of health outcomes: evidence from Bangladesh. Health Econ 2003, 12:415–419. | Bangladesh | Measles |
| Meij JJ, de Craen AJ, Agana J, Plug D, Westendorp RG. Low-cost interventions accelerate epidemiological transition in Upper East Ghana. Trans R Soc Trop Med Hyg 2009;103(February (2)):173–8. | Ghana | EPI |

**Cost-effectiveness studies**

| Citation | Vaccine | Significantly cost-effective ? |
| --- | --- | --- |
| Laxminarayan, R. Fighting antibiotic resistance: Can economic incentives play a role? Resources 2001, 143: 9-12. | Cholera | Varying |
| Knodel, J. and E. Vandewalle. Lessons from the Past - Policy Implications of Historical Fertility Studies. Population and Development Review 1979; 5:217-245. | Cholera | Varying |
| Dahan M, Tsiddon D. Demographic transition, income distribution, and economic growth. Journal of Economic Growth 1998, 3:29–52. | Cholera | Yes |
| Bloom DE, Canning D, Weston M. The value of vaccination. World Economics 2005, 6:15–39. | Hepatitis A | Yes |
| Beutels P, Edmunds WJ, Smith RD. Partially wrong? Partial equilibrium and the health economic analysis of public health emergencies of international concern. Health Econ 2008, 17:1317–1322. | Hepatitis B | Yes |
| Connolly MP, Postma MJ. Healthcare as an investment: Implications for an era of ageing populations. J Med Mark 2010;10(1):5-14. | Hepatitis B | No |
| Alsan M, Canning D, Bloom D E. The effect of population health on foreign direct investments. NBER Working Paper Series 2004; Working Paper 10596. | Hepatitis B | No |
| Bloom DE, Canning D, Weston M. The value of vaccination. World Economics 2005, 6:15–39. | HiB | No |
| Murray CJL et al. (2012). Disability-adjusted life years (DALYs) for 291 diseases and injuries in 21 regions, 1990—2010: a systematic analysis for the Global Burden of Disease Study 2010. The Lancet, 380 (9859): 2197-2223 | HiB | No |
| Murray CJ, Lopez AD. The Global Burden of Disease: a comprehensive assessment of mortality and disability from diseases, injuries and risk factors in 1990 and projected to 2020. Cambridge, MA, Harvard School of Public Health, (Global Burden of Disease and Injury Series, vol. I) | HiB | No |
| Koopmanschap MA, Rutten FF, van Ineveld BM, van Roijen L. The friction cost method for measuring indirect costs of disease. J Health Econ. 1995 Jun, 14(2):171-89. | Japanese encephalitis | Yes |
| Barrett S. Eradication versus control: the economics of global infection disease policy. Bulletin of the World Health Organization 2004; 82: 683-688. | Japanese encephalitis | Yes |
| Connolly M,  Constenla D. Assessing economic benefits for government and society attributed to malaria investment strategies: An exploratory analysis based on malaria vaccination. In Proceedings of The Multilateral Initiative on Malaria 2009: www.gmasoln.com/downloads/Malaria.pdf. | Malaria | Varying |
| Drummond M, Chevat C, LothgrenM. Do we fully understand the economic value of vaccines? Vaccine 2007; 25: 5945–5957. | Measles | Yes |
| Bärnighausen T, Bloom DE, Canning D, Friedman A, Levine O, O’Brien J, Privor-Dumm J, Walker D (2011). Rethinking the benefits and costs of childhood vaccination: the example of the Haemophilus influenza type b vaccine. Vaccine, 29(13): 2371-2380. | Measles | Yes |
| Goldie SJ, O’Shea M, Diaz M, Kim S-Y. Benefits, cost requirements and cost-effectiveness of the HPV16,18 vaccine for cervical cancer prevention in developing countries: policy implications. Reprod Health Matters 2008, 16(32):86–96. | PCV | Yes |
| World Health Organisation. The Global Burden of Disease: 2004 Update. World Health Organisation; Geneva Switzerland; 2008. | PCV | Varying |
| Lazano R et al. (2012).Global and regional mortality from 235 causes of death for 20 age groups in 1990 and 2010: a systematic analysis for the Global Burden of Disease Study 2010.The Lancet, 380(9859): 2095-2128. | PCV | Varying |
| Global Burden of Disease Study 2010. Global Burden of Disease Study 2010 Results by Cause 1990-2010. Seattle, United States: Institute for Health Metrics and Evaluation (IHME), 2012. | PCV | Varying |
| Simmerman JM, Lertiendumrong J, Dowell SF, Uyeki T, Olsen SJ, Chittaganpitch M, et al. The cost of influenza in Thailand. Vaccine 2006, 240:4417–26. | Polio | Varying |
| Berndt ER, Glennerster R, Kremer MR, Lee J, Levine R, Weizsäcker G, et al. Advance market commitments for vaccines against neglected diseases: estimating costs and effectiveness. Health Econ 2007, 16(5):491–511. | Polio | Varying |
| Duintjer Tebbens RJ, Pallansch MA, Cochi SL, Wassilak SGF, Linkins J, Sutter RW, et al. Economic analysis of the global polio eradication initiative. Vaccine 2010, 29(2):43–334. | Polio | Yes |
| Isakbaeva ET , Musabaev E, Antil L, Rheingans R, Juraev R, Glass RI, et al. Rotavirus disease in Uzbekistan: cost-effectiveness of a new vaccine. Vaccine 2007, 25(January (2)):373–80. | Rotavirus | Varying |
| Kim S-Y, Goldie SJ, Salomon JA. Cost-effectiveness of rotavirus vaccination in Vietnam. BMC Public Health 2009, 9(1):29. | Rotavirus | Varying |
| Rheingans Richard D, Antil L, Dreibelbis R, Podewils Laura J, Bresee Joseph S, Parashar Umesh D. Economic costs of rotavirus gastroenteritis and cost effectiveness of vaccination in developing countries. J Infect Dis 2009, 200(s1):S16–27. | Rotavirus | No |
| Atherly D, Dreibelbis R, Parashar Umesh D, Levin C, Wecker J, Rheingans Richard D. Rotavirus vaccination: cost effectiveness and impact on child mortality in developing countries. J Infect Dis 2009, 200(s1):S28–38. | Rotavirus | Yes |
| Berry SA, Johns B, Shih C, Berry AA, Walker DG. The cost-effectiveness of rotavirus vaccination in Malawi. J Infect Dis 2010, 202(Suppl.):S108–15. | Rotavirus | No |
| Clark AD, Walker DG, Mosqueira NR, Penny ME, Lanata CF, Fox-Rushby J, et al. Cost-effectiveness of rotavirus vaccination in Peru. J Infect Dis 2009, 200(Suppl. 1):S114–24. | Rotavirus | Yes |
| Constenla D, Velázquez FR, Rheingans RD, Antil L, Cervantes Y. Economic impact of a rotavirus vaccination program in Mexico. Rev Panam Salud Pública 2009, 25(6):481–90. | Rotavirus | Yes |
| Tate JE, Rheingans RD, O’Reilly CE, Obonyo B, Burton DC, Tornheim JA, et al. Rotavirus disease burden and impact and cost-effectiveness of a rotavirus vaccination program in Kenya. J Infect Dis 2009, 200(Suppl. 1): S76–84. | Rotavirus | Yes |
| Flem ET, Latipov R, Nurmatov ZS, Xue Y, Kasymbekova KT, Rheingans RD. Costs of diarrheal disease and the cost-effectiveness of a rotavirus vaccination program in Kyrgyzstan. J Infect Dis 2009, 200(November (Suppl. 1)):S195–202. | Rotavirus | Yes |
| Constenla DO, Linhares AC, Rheingans RD, Antil LR, Waldman EA, da Silva LJ. Economic impact of a rotavirus vaccine in Brazil. J Health Popul Nutr 2008, 26(4):388–96. | Rotavirus | Yes |
| Fischer TK, Anh DD, Antil L, Cat NDL, Kilgore PE, Thiem VD, et al. Health care costs of diarrheal disease and estimates of the cost-effectiveness of rotavirus vaccination in Vietnam. J Infect Dis 2005,192(10):1720–6. | Rotavirus | Varying |
| Wilopo SA, Kilgore P, Kosen S, Soenarto Y, Aminah S, Cahyono A, et al. Economic evaluation of a routine rotavirus vaccination programme in Indonesia. Vaccine 2009,27(November (Suppl. 5)):F67–74. | Rotavirus | Varying |

# III. Categorisation of relevant studies into potential benefits of vaccination

| **Category** | **References** |
| --- | --- |
| *A. Health-related benefits to vaccinated individuals (Narrow benefits)* | |
| A1. Health gains:  Reduction in morbidity and mortality  Examples: Cases averted, deaths averted, disability adjusted life years saved | Global Burden of Disease Study 2010. Global Burden of Disease Study 2010 Results by Cause 1990-2010. Seattle, United States: Institute for Health Metrics and Evaluation (IHME), 2012.  Jeuland M, Whittington D. Cost-benefit comparisons of investments in improved water supply and cholera vaccination programs. Vaccine 2009, 27:3109–3120.  Meij JJ, de Craen AJ, Agana J, Plug D, Westendorp RG. Low-cost interventions accelerate epidemiological transition in Upper East Ghana. Trans R Soc Trop Med Hyg 2009;103(February (2)):173–8.  Murray CJ, Lopez AD. The Global Burden of Disease: a comprehensive assessment of mortality and disability from diseases, injuries and risk factors in 1990 and projected to 2020. Cambridge, MA, Harvard School of Public Health, (Global Burden of Disease and Injury Series, vol. I)  World Health Organisation. The Global Burden of Disease: 2004 Update. World Health Organisation; Geneva Switzerland; 2008.  Lazano R et al. (2012).Global and regional mortality from 235 causes of death for 20 age groups in 1990 and 2010: a systematic analysis for the Global Burden of Disease Study 2010.The Lancet, 380(9859): 2095-2128.  Murray CJL et al. (2012). Disability-adjusted life years (DALYs) for 291 diseases and injuries in 21 regions, 1990—2010: a systematic analysis for the Global Burden of Disease Study 2010. The Lancet, 380 (9859): 2197-2223  **Cost per Daly averted from cost-effectiveness/cost-utility studies**^2^  Akumu AO, English M, Scott JA, Griffiths UK. Economic evaluation of delivering Haemophilus influenzae type b vaccine in routine immunization services in Kenya. Bull World Health Organ 2007, 85(July (7)):511–8.  Atherly D, Dreibelbis R, Parashar Umesh D, Levin C, Wecker J, Rheingans Richard D. Rotavirus vaccination: cost effectiveness and impact on child mortality in developing countries. J Infect Dis 2009, 200(s1):S28–38.  Berndt ER, Glennerster R, Kremer MR, Lee J, Levine R, Weizsäcker G, et al. Advance market commitments for vaccines against neglected diseases: estimating costs and effectiveness. Health Econ 2007, 16(5):491–511.  Berry SA, Johns B, Shih C, Berry AA, Walker DG. The cost-effectiveness of rotavirus vaccination in Malawi. J Infect Dis 2010, 202(Suppl.):S108–15.  Bishai D, Johns B, Lefevre A, Nair D. Cost effectiveness of measles eradication. World Health Organization 2010.  Broughton EI. Economic evaluation of Haemophilus influenzae type B vaccination in Indonesia: a cost-effectiveness analysis. J Public Health 2007, 29(4):441–8.  Chotivitayatarakorn P, Poovorawan Y. Cost-effectiveness of rotavirus vaccination as part of the national immunization program for Thai children. Southeast Asian J Trop Med Public Health 2010, 41:114–25.  Clark AD, Walker DG, Mosqueira NR, Penny ME, Lanata CF, Fox-Rushby J, et al. Cost-effectiveness of rotavirus vaccination in Peru. J Infect Dis 2009, 200(Suppl. 1):S114–24.  Constenla D, Velázquez FR, Rheingans RD, Antil L, Cervantes Y. Economic impact of a rotavirus vaccination program in Mexico. Rev Panam Salud Pública 2009, 25(6):481–90.  Constenla DO. Economic impact of pneumococcal conjugate vaccination in Brazil, Chile, and Uruguay. Rev Panam Salud Pública 2008, 24(2): 101–12.  Constenla DO, Linhares AC, Rheingans RD, Antil LR, Waldman EA, da Silva LJ. Economic impact of a rotavirus vaccine in Brazil. J Health Popul Nutr 2008, 26(4):388–96.  Cook J, Jeuland M, Whittington D, Poulos C, Clemens J, Sur D, et al. The cost- effectiveness of typhoid Vi vaccination programs: calculations for four urban sites in four Asian countries. Vaccine 2008, 26(November (50)):6305–16.  De la Hoz F, Alvis N, Narváez J, Cediel N, Gamboa O, Velandia M. Potential epidemiological and economical impact of two rotavirus vaccines in Colombia. Vaccine 2010, 28(22):3856–64.  Ding D, Kilgore P, Clemens J, Wei L, Zhi-Yi X. Cost-effectiveness of routine immunization to control Japanese encephalitis in Shanghai, China. Bull World Health Organ 2003, 81(5):334–42.  Duintjer Tebbens RJ, Pallansch MA, Cochi SL, Wassilak SGF, Linkins J, Sutter RW, et al. Economic analysis of the global polio eradication initiative. Vaccine 2010, 29(2):43–334.  Edejer TTT. Cost effectiveness analysis of strategies for child health in developing countries. Br Med J 2005, 331(7526):1177–80.  Fischer TK, Anh DD, Antil L, Cat NDL, Kilgore PE, Thiem VD, et al. Health care costs of diarrheal disease and estimates of the cost-effectiveness of rotavirus vaccination in Vietnam. J Infect Dis 2005,192(10):1720–6.  Flem ET, Latipov R, Nurmatov ZS, Xue Y, Kasymbekova KT, Rheingans RD. Costs of diarrheal disease and the cost-effectiveness of a rotavirus vaccination program in Kyrgyzstan. J Infect Dis 2009, 200(November (Suppl. 1)):S195–202.  Gessner BD, Sedyaningsih ER, Griffiths UK, Sutanto A, Linehan M, Mercer D, et al. Vaccine-preventable haemophilus influenza type B disease burden and cost-effectiveness of infant vaccination in Indonesia. Pediatr Infect Dis J 2008, 27(5):438–43.  Goldie SJ, O’Shea M, Campos NG, Diaz M, Sweet S, Kim S-Y. Health and economic outcomes of HPV 16,18 vaccination in 72 GAVI-eligible countries. Vaccine 2008, 26(32):4080–93.  Goldie SJ, O’Shea M, Diaz M, Kim S-Y. Benefits, cost requirements and cost-effectiveness of the HPV16,18 vaccine for cervical cancer prevention in developing countries: policy implications. Reprod Health Matters 2008, 16(32):86–96.  Griffiths UK, Botham L, Schoub BD. The cost-effectiveness of alternative polio immunization policies in South Africa. Vaccine 2006, 24(July (29–30)):5670–8.  Griffiths UK, Hutton G, Das Dores Pascoal E. The cost-effectiveness of introducing hepatitis B vaccine into infant immunization services in Mozambique. Health Policy Plan 2005, 20(January (1)):50–9.  Isakbaeva ET, Musabaev E, Antil L, Rheingans R, Juraev R, Glass RI, et al. Rotavirus disease in Uzbekistan: cost-effectiveness of a new vaccine. Vaccine 2007, 25(January (2)):373–80.  Jeuland M, Cook J, Poulos C, Clemens J, Whittington D. Cost-effectiveness of new-generation oral cholera vaccines: a multisite analysis. Value Health 2009, 12(6):899–908.  Khan MM. Economics of polio vaccination in the post-eradication era: should OPV-using countries adopt IPV? Vaccine 2008, 26(16):2034–40.  Kim S-Y, Goldie SJ, Salomon JA. Cost-effectiveness of rotavirus vaccination in Vietnam. BMC Public Health 2009, 9(1):29.  Kim S-Y, Lee G, Goldie SJ. Economic evaluation of pneumococcal conjugate vaccination in The Gambia. BMC Infect Dis 2010, 10:260.  Kim S-Y, Salomon J, Goldie S. Economic evaluation of hepatitis B vaccination in low-income countries: using cost-effectiveness affordability curves. Bull World Health Organ 2007, 85(11):833–42.  Ono S, Kurotaki T, Nakasone T, Honda M, Boon-Long J, Sawanpanyalert P, et al. Cost-effectiveness analysis of antiretroviral drug treatment and HIV-1 vaccination in Thailand. Jpn J Infect Dis 2006, 59:168–73.  Ortega O, El-Sayed N, Sanders JW, Abd-Rabou Z, Antil L, Bresee J, et al. Cost–benefit analysis of a rotavirus immunization program in the Arab Republic of Egypt. J Infect Dis 2009, 200(November (Suppl. 1)):S92–8.  Platonov AE, Griffiths UK, Voeykova MV, Platonova OV, Shakhanina IL, Chistyakova GG, et al. Economic evaluation of Haemophilus influenzae type b vaccination in Moscow, Russian Federation. Vaccine 2006, 24(March (13)):2367–76.  Podewils LJ, Antil L, Hummelman E, Bresee J, Parashar UD, Rheingans R. Projected cost-effectiveness of rotavirus vaccination for children in Asia. J Infect Dis 2005, 192(Suppl. 1):S133–45.  Prakash C. Crucial factors that influence cost-effectiveness of universal hepatitis B immunization in India. Int J Technol Assess Health Care 2003, 19(Winter (1)):28–40.  Rheingans Richard D, Antil L, Dreibelbis R, Podewils Laura J, Bresee Joseph S, Parashar Umesh D. Economic costs of rotavirus gastroenteritis and cost effectiveness of vaccination in developing countries. J Infect Dis 2009, 200(s1):S16–27.  Rheingans RD, Constenla D, Antil L, Innis BL, Breuer T. Potential cost-effectiveness of vaccination for rotavirus gastroenteritis in eight Latin American and Caribbean countries. Rev Panam Salud Publica 2007, 21(April (4)):205–16.  Sinha A, Constenla D, Valencia JE, O’Loughlin R, Gomez E, de la Hoz F, et al. Cost-effectiveness of pneumococcal conjugate vaccination in Latin America and the Caribbean: a regional analysis. Rev Panam Salud Pública 2008, 24(5):304–13.  Sinha A, Levine O, Knoll MD, Muhib F, Lieu TA. Cost-effectiveness of pneumococcal conjugate vaccination in the prevention of child mortality: an international economic analysis. Lancet 2007, 369(9559): 389–96.  Tate JE, Rheingans RD, O’Reilly CE, Obonyo B, Burton DC, Tornheim JA, et al. Rotavirus disease burden and impact and cost-effectiveness of a rotavirus vaccination program in Kenya. J Infect Dis 2009, 200(Suppl. 1): S76–84.  Tediosi F, Hutton G, Maire N, Smith TA, Ross A, Tanner M. Predicting the cost-effectiveness of introducing a pre-erythrocytic malaria vaccine into the expanded program on immunization in Tanzania. Am J Trop Med Hyg 2006, 75(2 Suppl.):131–43.  Touch S, Suraratdecha C, Samnang C, Heng S, Gazley L, Huch C, et al. A cost-effectiveness analysis of Japanese encephalitis vaccine in Cambodia. Vaccine 2010, 28(29):4593–9.  Vespa G, Constenla DO, Pepe C, Safadi MA, Berezin E, de Moraes JC, et al. Estimating the cost-effectiveness of pneumococcal conjugate vaccination in Brazil. Rev Panam Salud Pública 2009, 26(6):518–28.  Wilopo SA, Kilgore P, Kosen S, Soenarto Y, Aminah S, Cahyono A, et al. Economic evaluation of a routine rotavirus vaccination programme in Indonesia. Vaccine 2009,27(November (Suppl. 5)):F67–74. |
| A2. Healthcare cost savings:  Reduction in direct costs of healthcare borne by the public sector or private individuals  Examples: Costs saved | Stack ML, Ozawa S, Bishai DM, Mirelman A, Tam Y, Niessen L, Walker DG, Levine OS. Estimated economic benefits during the ‘Decade of Vaccines’ include treatment savings, gains in labor productivity. Health Aff 2011, 30:1021–1028.  **Outbreak prevention savings**  Apisarnthanarak A, Puthavathana P, Kitphati R, Auewarakul P, Mundy L. Outbreaks of Influenza A among nonvaccinated healthcare workers: implications for resource-limited settings. Infect Control Hosp Epidemiol 2008, 29(8):777–80.  Khan MM. Economics of polio vaccination in the post-eradication era: should OPV-using countries adopt IPV? Vaccine 2008, 26(16):2034–40.  Parent du Châtelet I, Gessner BD, da Silva A. Comparison of cost-effectiveness of preventive and reactive mass immunization campaigns against meningococcal meningitis in West Africa: a theoretical modeling analysis. Vaccine 2001, 19(25–26):3420–31.  **Willingness to pay studies**^2^  Cook J, Jeuland M, Maskery B, Lauria D, Sur D, Clemens J, et al. Using private demand studies to calculate socially optimal vaccine subsidies in developing countries. J Policy Anal Manage 2009;28(1):6–28.  Cropper M, Haile M, Lampietti J, Poulos C, Whittington D. The demand for a malaria vaccine: evidence from Ethiopia. J Dev Econ 2004;75:303–18.  Heinzen RR, Bridges JF. Comparison of four contingent valuation methods to estimate the economic value of a pneumococcal vaccine in Bangladesh. Int J Technol Assess Health Care 2008;24(Fall (4)):481–7.  Islam Z, Maskery B, Nyamete A, Horowitz MS, Yunus M, Whittington D. Private demand for cholera vaccines in rural Matlab, Bangladesh. Health Policy 2008;85(February (2)):184–95.  Jeuland M, Lucas M, Clemens J, Whittington D. Estimating the private benefits of vaccination against cholera in Beira, Mozambique: a travel cost approach. J Dev Econ 2010;91:310–22.  Kim D, Canh do G, Poulos C, Thoa le TK, Cook J, Hoa NT, et al. Private demand for cholera vaccines in Hue, Vietnam. Value Health 2008;11(January–February (1)):28–119.  Lucas ME, Jeuland M, Deen J, Lazaro N, MacMahon M, Nyamete A, et al. Private demand for cholera vaccines in Beira, Mozambique. Vaccine 2007;25(March (14)):2599–609.  Palanca-Tan R. The demand for a dengue vaccine: a contingent valuation survey in Metro Manila. Vaccine 2008;26(February (7)):914–23.  Sauerborn R, Gbangou A, Dong H, Przyborski JM, Lanzer M. Willingness to pay for hypothetical malaria vaccines in rural Burkina Faso. Scand J Public Health 2005;33(2):146–50.  Udezi WA, Usifoh CO, Ihimekpen OO. Willingness to pay for three hypothetical malaria vaccines in Nigeria. Clin Ther 2010;32(August (8)):1533–44.  Whittington D, Matsui-Santana O, Freiberger JJ, Van Houtven G, Pattanayak S. Private demand for a HIV/AIDS vaccine: evidence from Guadalajara, Mexico. Vaccine 2002;20(June (19–20)):2585–91.  Whittington D, Suraratdecha C, Poulos C, Ainsworth M, Prabhu V, Tangcharoensathien V. Household demand for preventive HIV/AIDS vaccines in Thailand: do husbands’ and wives’ preferences differ? Value Health 2008;11(September–October (5)):965–74.  Whittington D, Sur D, Cook J, Chatterjee S, Maskery B, Lahiri M, et al. Rethinking cholera and typhoid vaccination policies for the poor: private demand in Kolkata, India. World Dev 2009;37(2):399–409. |
| *B. Productivity-related benefits* | |
| B1. Productivity gains related to care:  Reduction in lost days of work due to sickness or caring for a sick patient  Examples: Value of productivity gained |  |
| B2. Productivity gains related to short-term outcomes:  Reduction in lost days of work  Examples: Value of productivity gained, lifetime earnings | Koopmanschap MA, Rutten FF, van Ineveld BM, van Roijen L. The friction cost method for measuring indirect costs of disease. J Health Econ. 1995 Jun, 14(2):171-89. |
| B3. Productivity gains related to long term outcomes:  Increased lifetime productivity because better health improves cognition, educational attainment and physical strength  Examples: Educational outcomes, cognitive outcomes, lifetime earnings | Barham T, Calimeris L. Long-term effects of family planning and child health interventions on adolescent cognition: Evidence from Matlab in Bangladesh. Working paper. University of Colorado: Health and Society program; 2008.  Bärnighausen T, Bloom DE, Canning D, Friedman A, Levine O, O’Brien J, Privor-Dumm J, Walker D (2011). Rethinking the benefits and costs of childhood vaccination: the example of the Haemophilus influenza type b vaccine. Vaccine, 29(13): 2371-2380.  Bloom DE, Canning D, Weston M. The value of vaccination. World Economics 2005, 6:15–39.  Bloom D E, Canning D, Seiguer E. The effect of vaccination on children's physical and cognitive development in the Philippines. Applied Economics 2012; 44: 2777–2783.  Canning D, Razzaque A, Driessen J, Walker D G, Streatfield P K, Yunus M. The effect of maternal tetanus immunization on children’s schooling attainment in Matlab, Bangladesh: follow-up of a randomized trial. Social Science & Medicine 2011; 72: 1429-1436.  Connolly M, Constenla D. Assessing economic benefits for government and society attributed to malaria investment strategies: An exploratory analysis based on malaria vaccination. In Proceedings of The Multilateral Initiative on Malaria 2009: www.gmasoln.com/downloads/Malaria.pdf.  Connolly MP, Postma MJ. Healthcare as an investment: Implications for an era of ageing populations. J Med Mark 2010;10(1):5-14.  Driessen J, Razzaque A, Walker D, Canning D. The effect of childhood measles vaccination on school enrolment in Matlab, Bangladesh. PGDA Working Paper 2011; 81 <http://www.hsph.harvard.edu/pgda/working.htm>  Kumar S. Childhood immunization, mortality and human capital accumulation: Micro- evidence from India. Working paper. University of Houston: Harvard Centre for Population and Development Studies; 2009. http://mpra.ub.uni-muenchen.de/31806/.  Ozawa S, Stack ML, Bishai DM, Mirelman A, Friberg IK, Niessen L, Walker DG, Levine OS. During the ‘Decade of Vaccines’ the lives of 6.4 million children valued at $231 billion could be saved. Health Aff 2011, 30:1010–1020.  Simmerman JM, Lertiendumrong J, Dowell SF, Uyeki T, Olsen SJ, Chittaganpitch M, et al. The cost of influenza in Thailand. Vaccine 2006, 240:4417–26.  Stack ML, Ozawa S, Bishai DM, Mirelman A, Tam Y, Niessen L, Walker DG, Levine OS. Estimated economic benefits during the ‘Decade of Vaccines’ include treatment savings, gains in labor productivity. Health Aff 2011, 30:1021–1028.  Tebbens RJD, Pallansch MA, Cochi SL, Wassilak SGF, Linkins J, Sutter RW, Aylward RB, Thompson KM. Economic analysis of the global polio eradication initiative. Vaccine 2011, 29:334–343. |
| B4. Productivity gains related to household behaviour:  Economic improvements due to changes in household choices such as fertility and consumption/saving as a result of improved child health and survival  Examples: Productivity, female labour participation, household investment per child, dependency ratio | Knodel, J. and E. Vandewalle. Lessons from the Past - Policy Implications of Historical Fertility Studies. Population and Development Review 1979; 5:217-245.  **Lifetime benefit models**  Bloom DE, Canning D, Weston M. The value of vaccination. World Economics 2005, 6:15–39.  Connolly M, Constenla D. Assessing economic benefits for government and society attributed to malaria investment strategies: An exploratory analysis based on malaria vaccination. In Proceedings of The Multilateral Initiative on Malaria 2009: www.gmasoln.com/downloads/Malaria.pdf. |
| *C. Community externalities* | |
| C1. Ecological effects:  Health improvements in unvaccinated community members as a result of ecological effects such as herd immunity and reduced antibiotic usage.  Examples: Indirect vaccine protection, prevalence of antibiotic resistance | Barrett S. Eradication versus control: the economics of global infection disease policy. Bulletin of the World Health Organization 2004; 82: 683-688.  Beutels P, Edmunds WJ, Smith RD. Partially wrong? Partial equilibrium and the health economic analysis of public health emergencies of international concern. Health Econ 2008, 17:1317–1322.  Cook J, Jeuland M, Maskery B, Lauria D, et al. Using private demand studies to calculate socially optimal vaccine subsidies in developing countries. J Policy Anal Manage 2009, 28:6–28.  Drummond M, Chevat C, LothgrenM. Do we fully understand the economic value of vaccines? Vaccine 2007; 25: 5945–5957.  Giglio ND, Cane AD, Micone P, Gentile A. Cost-effectiveness of the CRM-based 7- valent pneumococcal conjugated vaccine (PCV7) in Argentina. Vaccine 2010, 28:2302– 2310.  Jeuland M, Cook J, Poulos C, Clemens J, Whittington D. Cost-effectiveness of new- generation oral cholera vaccines: A multisite analysis. Value Health 2009, 12:899–908.  Jeuland M, Lucas M, Clemens J, Whittington D. A Cost–Benefit Analysis of Cholera Vaccination Programs in Beira, Mozambique. World Bank Econ Rev 2009, 23:235–267.  Jeuland M, Whittington D. Cost-benefit comparisons of investments in improved water supply and cholera vaccination programs. Vaccine 2009, 27:3109–3120.  Kim SY, Lee G, Goldie SJ. Economic evaluation of pneumococcal conjugate vaccination in The Gambia. BMC Infect Dis 2010, 10:260–278.  Laxminarayan, R. Fighting antibiotic resistance: Can economic incentives play a role? Resources 2001, 143: 9-12.  Lopez E, Debbag R, Coudeville L, Baron-Papillon F, Armoni J. The cost-effectivness of universal vaccination of children against hepatitis A in Argentina: results of a dynamic health-economic analysis. J Gastroenterology 2007, 42:152–160.  Vespa G, Constenla DO, Pepe C, Safadi MA, Berezin E, Moraes JC, Campos CAH, Araujo DV, Andrade ASS. Estimating the cost-effectiveness of pneumococcal conjugate vaccination in Brazil. Pan Am J Public Health 2009, 26:518–528. |
| C2. Equity:  More equal distribution of health outcomes  Examples: Distribution of health outcomes | Bawah AA, Phillips JF, Adjuik M, Vaughan-Smith M, Macleod B, Binka FN. The impact of immunization on the association between poverty and child survival: Evidence from Kassena-Nankana district of northern Ghana. Scand J Public Health 2010, 38:95–103.  Bishai D, Koenig M, Khan MA. Measles vaccination improves the equity of health outcomes: evidence from Bangladesh. Health Econ 2003, 12:415–419. |
| C3. Financial sustainability:  Improved financial sustainability of health care programs as a result of synergies with vaccination programs and/or stimulation of private demand.  Examples: Financial benefits, private demand estimates | **Opportunities for cost-sharing with other health and social interventions**  Jeuland M, Whittington D. Cost-benefit comparisons of investments in improved water supply and cholera vaccination programs. Vaccine 2009, 27:3109–3120.  Niessen L, ten Hove A, Hilderink H, Weber M, Mulholland K, Ezzati M. Comparative impact assessment of child pneumonia interventions. Bull World Health Organ 2009, 87:472–480.  Tebbens RJD, Pallansch MA, Cochi SL, Wassilak SGF, Linkins J, Sutter RW, Aylward RB, Thompson KM. Economic analysis of the global polio eradication initiative. Vaccine 2011, 29:334–343.  **Creating demand for vaccines and potential for partial cost recovery**  Cook J, Jeuland M, Maskery B, Lauria D, et al. Using private demand studies to calculate socially optimal vaccine subsidies in developing countries. J Policy Anal Manage 2009, 28:6–28.  Kim D, Canh DG, Poulos C, Thoa LTK, et al. Private demand for cholera vaccines in Hue, Vietnam. Value Health 2008, 11:119–128.  Lucas MES, Jeuland M, Deen J, Lazaro N, et al. Private demand for cholera vaccines in Beira, Mozambique. Vaccine 2007, 25:2599–2609.  Ozawa S, Stack ML, Bishai DM, Mirelman A, Friberg IK, Niessen L, Walker DG, Levine OS. During the ‘Decade of Vaccines’ the lives of 6.4 million children valued at $231 billion could be saved. Health Aff 2011, 30:1010–1020.  Palanca-Tan R. The demand for a dengue vaccine: A contingent valuation survey in metro Manila. Vaccine 2008, 26:914–923. 2008.  Sauerborn R, Gbanjou A, Dong H, Przyborski JM, Lancer M. Willingness to pay for hypothetical malaria vaccines in rural Burkina Faso. Scand J Public Health 2005, 33:146–150 (2005).  Udezi WA, Usifoh CO, Ihimekpen OO. Willingess to pay for three hypothetical malaria vaccines in Nigeria. Clin Ther 2010, 32:1533–1544.  Whittington D, Suraratdecha C, Poulos C, Ainsworth M, Prabhu V, Tangcharoensathien V. Household demand for preventive HIV/AIDS vaccines in Thailand: Do husbands’ and wives’ preferences differ? Value Health 2008, 11:965–974.  Whittington D, Sur D, Cook J, Chatterjee S, et al. Rethinking cholera and typhoid vaccination policies for the poor: Private demand in Kolkata, India. World Dev 2009, 37:399–409. |
| C4. Macroeconomic impact:  Changes in the national economy or individual sectors of the economy.  Examples: Gross domestic product, production by economic sector | Alsan M, Canning D, Bloom D E. The effect of population health on foreign direct investments. NBER Working Paper Series 2004; Working Paper 10596.  <http://www.nber.org/papers/w10596>  Beutels P, Edmunds WJ, Smith RD. Partially wrong? Partial equilibrium and the health economic analysis of public health emergencies of international concern. Health Econ 2008, 17:1317–1322.  Dahan M, Tsiddon D. Demographic transition, income distribution, and economic growth. Journal of Economic Growth 1998, 3:29–52.  Novaes H, Luna E, Goldbaum M, Kilsztajn S, Rossbach A, de la Rocha Carval- heiro J. The potential demand for an HIV/AIDS vaccine in Brazil. World Bank policy research working paper. The World Bank; 2002 p. 1–30. |
